# Evaluating the influencing factors of urbanization in the Xinjiang Uygur Autonomous Region over the past 27 years based on VIIRS-DNB and DMSP/OLS nightlight imageries

**DOI:** 10.1371/journal.pone.0235903

**Published:** 2020-07-22

**Authors:** Xueping Li, Xiaodong Yang, Lu Gong

**Affiliations:** 1 College of Resources and Environment Science, Xinjiang University, Urumqi, China; 2 Department of Geography & Spatial Information Technology, Ningbo University, Ningbo, China; 3 Key Laboratory of Oasis Ecology, Xinjiang University, Urumqi, China; Institute for Advanced Sustainability Studies, GERMANY

## Abstract

The Xinjiang Uygur Autonomous Region is the core economic area of the “Silk Road Economic Belt”. The urbanization of this region plays a highly important role in economic and cultural communications between China, Central Asia and Europe. However, the influencing factors of urbanization in this region remain unclear. In this study, we presented a new modified thresholding method to extract the urban built-up areas from two nightlight remote sensing data sources, *i*.*e*., the DMSP/OLS and VIIRS/DNB nightlight imageries. Then, geographical detectors and hierarchical partitioning analysis were used to test the influences of anthropogenic and geographic environmental factors on urbanization. Our results showed that the relative error between the actual and the extracted urban built-up areas calculated using our method ranged from -0.30 to 0.27 in two biggest sample cities (Urumqi and Karamay) over the last 27 years. These errors were lower than those calculated by using the traditional method (-0.66 ≤ relative error ≤ -0.11). The expansion of urban built-up areas was greater in the northern regions than the southern regions of Xinjiang, as well as was greater in large cities than small and medium-sized cities. The influence of anthropogenic factors on urbanization has continually decreased over the past 27 years, while the influence of geographical environmental factors has increased. Among all influencing factors, fixed asset investment, topographic position index and per capita possession of water resources have the high contributions on urbanization, accounting for 18.75%, 15.62% and 14.18% of the variance of urbanization, respectively. Here, we provided a new method for studying urbanization by using remote sensing data. Our results are helpful for understand the driving factors of urbanization, and they provide guidance for the sustainable economic development of the Xinjiang Uygur Autonomous Region.

## Introduction

City is the political, economic, social and cultural center of a given region, hence, its urbanization level relates with living quality and the sustainable development of local human beings [1,2]. Urbanization is an inevitable result of country development, through which traditional agricultural rural areas transform into a modern society dominated by manufacturing and service industries [3,4]. Urbanization is considered as one of the major contributors to global change, with its parallel increase in population size and change in human lifestyle [5,6]. Urbanization is expected to continue to support an increasing in situ population, which will therefore expand the city scale in future, and ignite a series of questions on the sustainability of social development / urbanization problems (*i*.*e*., food and water shortages, environmental degradation and a rise in unemployment) [6–9]. Previous studies have suggested that the solution of urbanization problems is a great challenge for government and academics due to the complexity and the variability of the influencing factors [8,9]. Evaluating the influencing factors of urbanization has been considered one of the most important ways in solving urbanization problems [10–12].

The expansion of urban built-up area is the most typical characteristic of urbanization [12], hence, which is usually used a base data to calculate urbanization rate and analyze the influencing factors of urbanization in previous studies [11,12]. However, although there are many traditional statistical methods, such as spatial autocorrelation and the coupled coordination model, were used to obtain urban built-up area, their effectiveness is relatively low due to the shortcomings in consecutive expression and the small accuracy [13,14]. In recent decades, the development of remote sensing technology, especially the use of nightlight remote sensing imageries that display the change in urban built-up area anytime and anyplace through photographs, has provided new insights by studying the progression of urbanization [15,16]. In nightlight remote sensing imagery, urban built-up areas are signified by a bright patch, which obviously distinguishes from non-urban areas [17]. Utilization of nightlight remote sensing imagery improves the extraction accuracy of urban built-up area by lowering confusion between urban and non-urban areas [15–18]. The usage of nightlight remote sensing imageries is considered a most effective method to obtain urban built-up area [16].

The natural limitation of nightlight remote sensing imagery on continuous extraction of urban built-up area is satellite run time [19]. At present, DMSP/OLS and VIIRS/DNB nightlight imageries are the most common data sources in scientific research. DMSP/OLS imageries were acquired by the Defense Meteorological Satellite from 1992 to 2013, while VIIRS/DNB imageries have been captured by NASA-NOAA Suomi National Polar-orbiting satellite since 2012 [20]. Due to difference in run time, DMSP/OLS or VIIRS/DNB imageries cannot be used individually to extract a continuous picture of urban built-up area since 1992 [20,21]. A combination of DMSP/OLS and VIIRS/DNB nightlight imageries may be a solving ways for above issues [20,21]. However, due to differences in satellite sensor and digital number (DN) values, the DMSP/OLS and VIIRS/DNB nightlight imageries extracted urban built-up area in different ways [22]. In order to be able to display the urban built-up area’s change through a combination of these images, it is necessary to develop a new method that can be used for both VIIRS/DNB and DMSP/OLS nightlight imageries.

Anthropogenic activities such as population migration and socio-economic activities are considered as the main factors affecting urbanization [6,23]. However, recent studies have discovered that geographic environmental factors also can affect urbanization probably because it has an obvious influence on human productive activities [13,24,25]. Further, geographic environmental factors affect people's perception of life quality, then promoting population migration and urbanization [6]. For example, an area with a mild temperature and gentle terrain tends to attract people to settle down. In addition, the relative impact of anthropogenic activities and geographic environmental factors on urbanization might be different, also which may be change over time [12]. However, our current understanding of their contributions on urbanization remains unclear.

In this study, we collected two kinds of nightlight remote sensing data, *i*.*e*., DMSP/OLS and VIIRS/DNB nightlight imageries, that document the Xinjiang Uygur Autonomous Region over the past 30 years. The purposes of this study are: (1) to develop a new method for using both VIIRS/DNB and DMSP/OLS nightlight imageries in tandem to extract urban built-up area; (2) to explore the influencing factors of urbanization. Our results will help reveal the internal mechanisms of urbanization and provide a reference for the sustainable development and scientific management of urban.

## Materials and methods

### Study area

**Fig 1:**
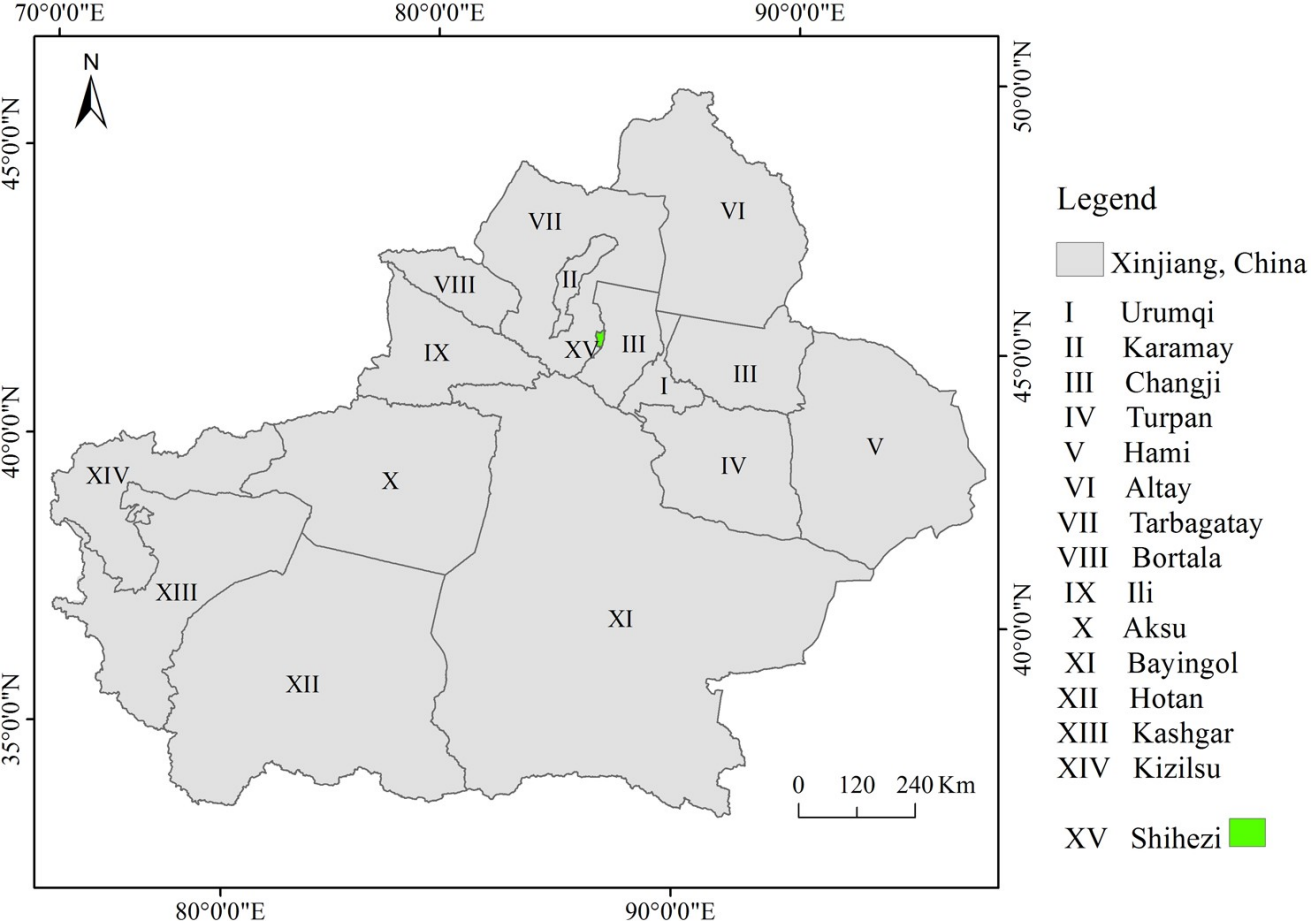
The composition of Xinjiang Uygur Autonomous Region. The vector map of Xinjiang Uygur Autonomous Region is downloaded from The Gateway to Astronaut Photography of Earth website (https://eol.jsc.nasa.gov/SearchPhotos/). Because the downloaded maps from this website are free and open to scholars around the world, our study do not need to supply a copyright statement.

The Xinjiang Uygur Autonomous Region (abbreviation is Xinjiang) is located in northwest China (E73°40′~96°23′, N34°25′~49°10′), covering an area of over 1.66×10^6^ km^2^ (Fig 1). The typical geomorphic structural units of the territory, from high latitudes to low latitudes, are: Altai Mountains, Junggar Basin, Tianshan Mountains, Tarim Basin and Kunlun Mountains. The terrain is undulating, and the elevation ranges from -155 m to 8611 m. In the temperate continental arid climate zone, the average annual precipitation is about 155 mm. Because it shares borders with many countries and it is the last marketplace in northwest China along the “Silk Road Economic Belt”, Xinjiang is considered the core area for economic and cultural exchange between China, Central Asia and Europe [25]. Xinjiang has 15 prefecture-levels regions (Fig 1). The vector map of Xinjiang Uyghur Autonomous Region was downloaded from The Gateway to Astronaut Photography of Earth website (https://eol.jsc.nasa.gov/SearchPhotos/). Because the downloaded maps from this website are free and open to scholars around the world, our study do not need to supply a copyright notice.

### Nightlight remote sensing imageries

DMSP/OLS and VIIRS/DNB nightlight imageries were all downloaded from the American National Geophysical Data Center (http://ngdc.noaa.gov.html). Previous studies have indicated that the OLS sensor was easily affected by atmospheric and light scattering, thus resulting in the discontinuous DN values of each pixel that are not comparable between different years. Therefore, the DMSP/OLS data sources must be corrected. In this study, using a calibration model of Elvidge et al. [26,27] and Fan et al. [28] to correct DN values (Eq 1). Unlike with the DMSP/OLS nightlight imageries, the orbit corrected procedure and the low-light detection capability synthesized VIIRS/DNB data to extract urban area without calibration. In this study, based on the geographical location, the monthly average light radiation of VIIRS/DNB synthesized data in the northern hemisphere from 2012 to 2018 was selected as the objective imagery. Because DN values of each pixel in VIIRS/DNB imageries could be affected by snow in winter, the average of the monthly average light radiation of VIIRS/DNB synthesized data was compiled using images from March to October to create a final image each year.

$$DN_{adjusted}=C_{0}+C_{1}\times DN+C_{2}\times DN^{2}$$ (1)

### Extraction of urban built-up areas from nightlight imageries

Extracting urban built-up areas from nightlight imageries is the precondition for calculating urbanization index and urbanization rate. Researchers have constructed some innovative methods, such as the experiential thresholding method and the statistical comparative method, to extract urban built-up areas from nightlight imageries [29–31]. However, the application of the experiential thresholding method is limited at a large scale for the following reasons: (1) the threshold for distinguishing the urban built-up areas from non-urban areas is determined through training involving a large number of repeated experiments. The number of experiments has to increase with the size of urban area; (2) the use of the experiential thresholding method to extract urban areas always ignores the temporal change of urbanization. The statistical comparative method also is limited at larger scales because it is based on the comparison between the detailed historical statistical data and the extracted urban areas [31]. At a larger scale, the detailed historical statistical data of urban areas is difficult to obtain. Therefore, it is necessary to develop a simple and effective method to extract urban built-up areas. In this study, we propose a new modified thresholding method that combines the advantages of both the traditional empirical thresholding and the statistical comparative methods. Because the threshold is based on training in the few sample cities that have detailed historical statistical urban areas in each year from 1992–2018, our modified thresholding method obtains a reasonable threshold while also reducing the error of urban area extraction at large scales. Here the actual detailed statistical urban built-up areas of the sample cities were obtained from China’s Urban Construction Statistical Yearbooks. The constructive process of our method can be described as follows:

1. In a given year (*j*), the urban built-up area (*S*_*i*_) of *X*_*i*_ cities with actual detailed statistical urban built-up areas are selected as reference indicators;
2. Based on *S*_*i*_, the statistical comparative method is used to calculate the optimal threshold (*v*_*i*_) of each selected city. Here threshold is a given DN values;
3. After *v*_*i*_ is used to obtain the extracted urban built-up area (*A*_*i*_) of each selected city, then *A*_*i*_ and *S*_*i*_ are used to obtain the extracted total urban built-up area (*TA*_*i*_) and the statistical total urban built-up area of all selected cities;
4. The difference between *TA*_*i*_ and the statistical total urban built-up area was compared. The *v*_*i*_ with the smallest difference between *TA*_*i*_ and the statistical total urban built-up area was set as the most reasonable threshold for given year *j*;
5. *v*_*i*_ was used to extract the total urban built-up area of all cities of the whole region at given year *j*;
6. The above steps were repeated for each year from 1992 to 2018 to extract the total urban built-up area of all cities in the whole region.

Compared with DMSP/OLS imageries, the higher DN values in VIIRS/DNB imageries reduce the accuracy of threshold classification [32,33]. In other words, due to differences in the *DN* values, the extracted built-up urban area might not be consistent between DMSP/OLS and VIIRS/DNB imageries, even when using our modified thresholding method. Thus, in this study, we removed deserts, lakes, high altitude areas and NDVI >0.45 areas from VIIRS/DNB imagery in 2012 and 2013 to improve the consistency of the extracted built-up urban areas between two data sources [34]. The details are listed in the supplementary materials of S1 Data.

### Urbanization index and urbanization rate

Previous studies have shown that the range of *DN* values (*i*.*e*., an area with a *DN* values >0) in nightlight imageries correlates highly with population and *GDP* [31,32]. The composition of *DN* values and its range can be used to construct urbanization index and urbanization rate (Eqs 2 and 3), where *RUI* and *RUR* were urbanization index and urbanization rate, respectively; *DN*_*i*_ and *N*_*i*_ represented the *DN* values and the pixel number at *i* level of *DN* values; *DN*_*min*_ *and DN*_*max*_ represented optimal threshold and maximum of the *DN* values in the study area, respectively. Optimal threshold (*DN*_*min*_) was obtained using our modified thresholding method. *S*_*N*_ was the total urban built-up area in a given city that extracted from nightlight imageries. *ΔRUI* was the difference between the urbanization index of a given place for several consecutive years; *ΔT* was the time interval over consecutive years. Because run time differed between the DMSP/OLS and VIIRS/DNB nightlight imageries, *RUI* and *RUR* were calculated using DMSP/OLS nightlight imagery for 1992 to 2012 and using VIIRS/DNB data for 2013 to 2018.

$$RUI=\frac{\sum_{i=\min}^{i-\max}DN_{i}\times N_{i}}{S_{N}}$$ (2)

$$RUR=\frac{\Delta RUI}{\Delta T}$$ (3)

### The influencing factors of urbanization

Nine factors were analyzed for their influence on urbanization, which can be classified into two types: anthropogenic and geographic environmental factors [24,25,35–37]. Anthropogenic factors included population density, gross domestic product (GDP), manufacturing and producer services proportion (MPSP) and fixed asset investment (FAI). Geographic environmental factors included degree of relief, topographic position index, slope, per capita possession of water resources and average annual temperature of land surface. Anthropogenic factors and per capita possession of water resources were collected from the China City Statistical Yearbook, the China Urban Construction Statistical Yearbook, and the Xinjiang Statistical Yearbook and the Statistical Yearbooks of various prefecture-levels in Xinjiang. The degree of relief, topographic position index and slope were calculated from the 30 m resolution DEM data published by the China Geospatial Data Cloud Website (http://www.gscloud.cn/). Average annual temperature of land surface was obtained from the MODIS 'MOD11-C3 product data from the Earthdata Website (https://search.earthdata.nasa.gov/search).

### Statistical analysis of the influencing factors of urbanization

Geographical detectors were used to estimate the relationship between urbanization index (*RUI*) and their underlying influencing factors [38]. The magnitude of influence of factors on *RUI* (*q*_*D*,*U*_) ranged from 0 to 1. *q*_*D*,*U*_ = 0 indicated that the influencing factor had no connection with *RUI*, whereas *q*_*D*,*U*_ = 1 suggested that *RUI* was completely determined by the influencing factor. When 0< *q*_*D*,*U*_ < 1, the large value of *q*_*D*,*U*_ suggested greatly influence of factor on *RUI* [38]. Hierarchical partitioning analysis was used to analyze the contribution of each influencing factor on *RUI*. Here, contribution is the proportion of each independent variable from the goodness-of-fit measures across all variable combinations in a hierarchy.

## Results and discussion

### The feasibility of our modified thresholding method in the extraction of urban built-up areas from nightlight imageries

**Fig 2:**
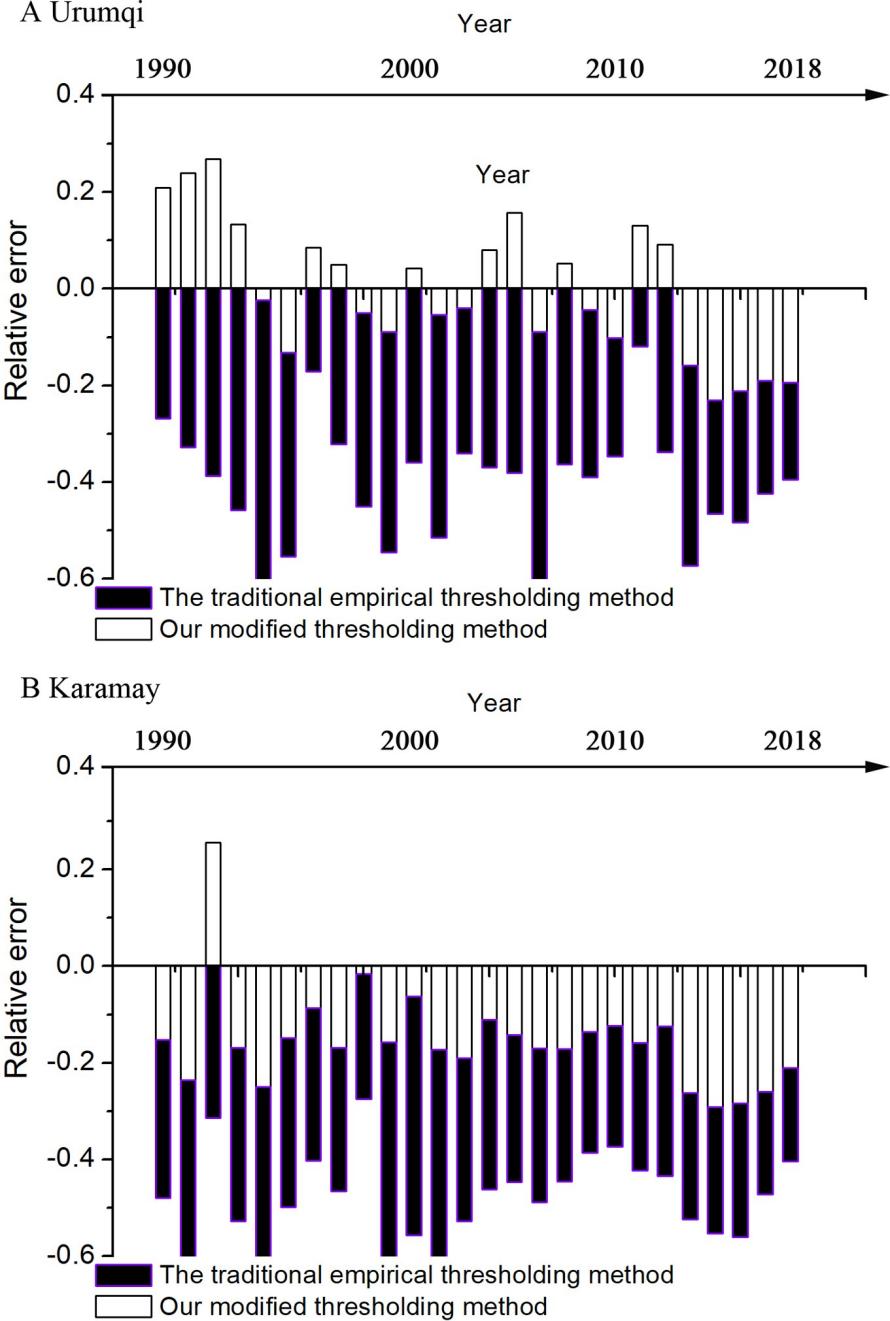
Relative error between the extracted and the actual urban built-up areas in Urumqi and Karamay of the Xinjiang Uygur Autonomous Region.

In this study, two biggest cities in Xinjiang, *i*.*e*., Urumqi and Karamay, were selected as samples for testing technical feasibility of our modified thresholding method. The results showed that the relative error between the extracted and the actual urban built-up areas over the last 27 years ranged from -0.30 to 0.27 when using our modified thresholding method, which was lower than that obtained using the traditional empirical thresholding method (-0.66 ≤relative error≤-0.11) (Fig 2). Additionally, the Paired T test showed that our modified thresholding method had a significantly lower relative error than the traditional empirical thresholding method in both Urumqi and Karamay (Table 1). This suggests that our modified thresholding method is highly effective at extracting urban built-up areas from nightlight imageries. Here we used the experiential thresholding method as a reference to test the feasibility of our method because it has previously been considered the most reliable method of extracting built-up area from two nightlight imageries [30,31].

**Table 1:** Difference in the relative error between our modified thresholding method and the traditional empirical thresholding method tested by the Paired T-test.

City	The traditional empirical thresholding method	Our modified thresholding method	*T*	*P*-value
Urumqi	-0.35±0.12	-0.003±0.14	-9.45	<0.001
Karamay	-0.32±0.08	-0.15±0.11	-6.02	<0.001

The improvement in effectiveness of our modified thresholding method in the extraction of urban build-up area from nightlight imageries has an obvious difference between DMSP/OLS and VIIRS/DNB data resources (Fig 2). For DMSP/OLS nightlight imagery, the relative error of our modified thresholding method was significantly lower than that of the traditional empirical thresholding method in the two sample cities. In contrast, there was no significant difference in relative error between our modified and traditional thresholding methods in the VIIRS/DNB data resource. These indicated that improvement of our method in the extraction of urban built-up area was high in DMSP/OLS than that in VIIRS/DNB nightlight imagery. This may be due to the fact that the redundant information of high-quality VIIRS/DNB imagery muddled the extraction of urban built-up area [39].

### Changes in urban built-up area over the last 27 years

**Fig 3:**
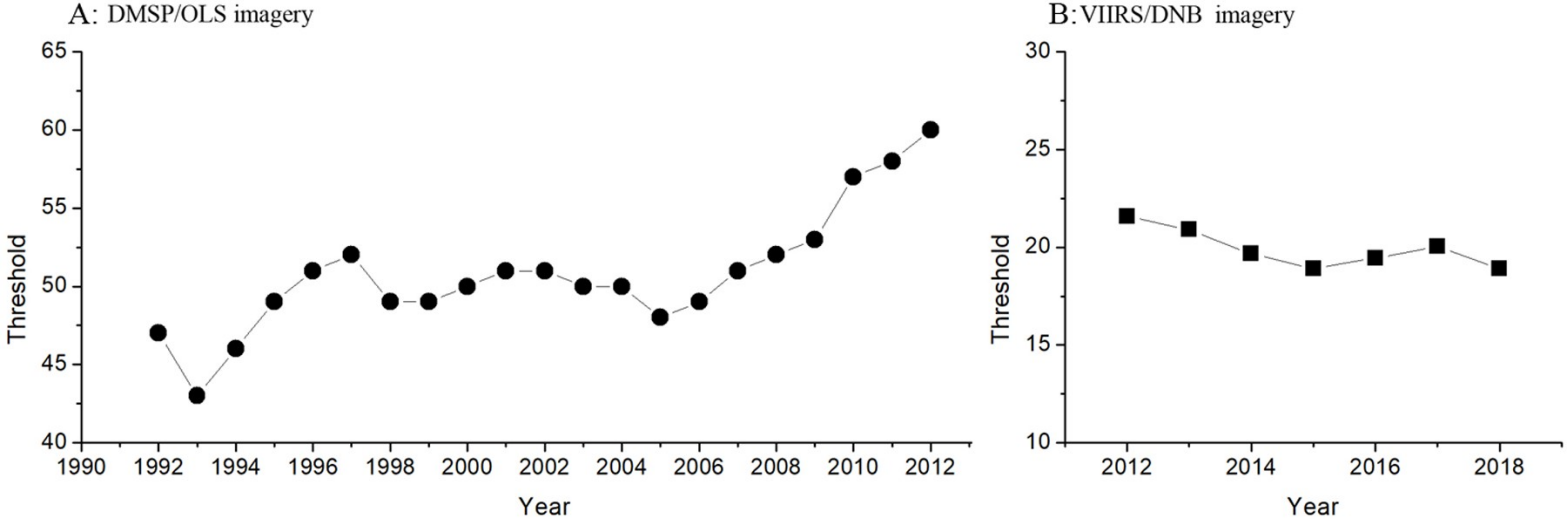
The optimal threshold for the extraction of built-up urban area form DMSP/OLS and VIIRS/DNB nightlight imageries over the past 27 years.

**Fig 4:**
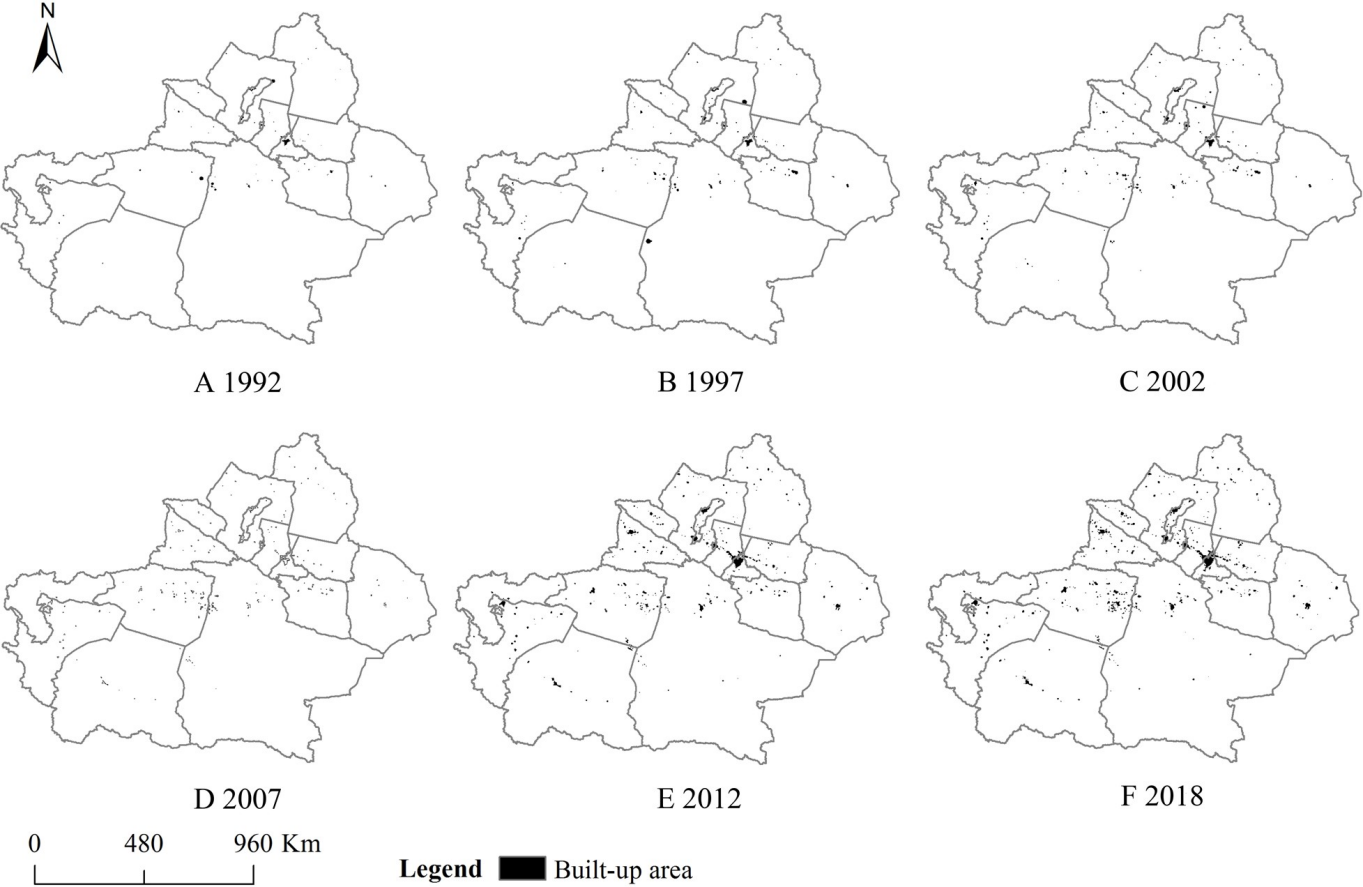
Changes in urban built-up areas in the Xinjiang Uygur Autonomous Region over the past 27 years. Copyright statement as suggested in Fig 1.

In this study, four cities with detailed historical statistical urban built-up areas were selected as samples to calculate the optimal thresholds from 1992 to 2018 (Fig 3). Then, urban built-up areas of whole Xinjiang were extracted from two nightlight imageries based on these optimal thresholds. In order to reduce the confusion and the expressive repetition, as suggested in the China Five-Year Plan and previous studies, six years, *i*.*e*., 1992, 1997, 2002, 2007, 2012 and 2018, were selected as benchmarks in which to indicate changes of urban built-up area. Our results showed that number and area of urban built-up areas increased continuously over the past 27 years (Table 2 and Fig 4). The magnitude of urban built-up areas had an obvious difference between northern and southern regions of Xinjiang (Table 2 and Fig 4). More specifically, northern region showed greater increases in urban built-up areas compared with southern. The gap in the number and area of urban built-up areas between northern and southern regions increased with passing year (Table 2 and Fig 4). Two urban agglomerations have formed around Urumqi (I) and Changji (III) city in the northern region (represented by the large shape of built-up area in multiple geographic areas), whereas no big urban agglomeration appeared in southern region (Table 2 and Fig 4). These indicated that urbanization process was more rapid in northern than southern regions. This is likely due to the differences in transportation, geographic environment, production mode and mineral reserves [25,40–43]. For example, southern region has historically been dominated by agriculture, while northern region was mostly relied on industry. The advantage in production mode quickened urbanization process in the northern regions [40]. Also, since northern region owned higher developed intensity of mineral resources and more convenient transportation than southern region [41,42], Chinese government established many industrial cities in northern region to exploit mineral resources, thus promoting urbanization [43].

**Table 2:** Changes in expansion intensity, the number and area of spot of urban built-up areas in the Xinjiang Uygur Autonomous Region over the past 27 years. Roman numerals are the serial number of different cities. I, II, III, IV, V, VI, VII, VIII, IX, X, XI, XII, XIII, XIV and XV represent Urumqi, Karamay, Changji Hui Autonomous Prefecture, Turpan, Hami, Altay, Tacheng Prefecture, Bortala Mongolian autonomous prefecture, Ili Kazakh Autonomous Prefecture, Aksu, Bayingolin Mongol Autonomous Prefecture, Hotan, Kashgar, Kizilsu Kirghiz Autonomous Prefecture and Shihezi, respectively.

Year	Number and area of spot	I	II	III	IV	V	VI	VII	VIII	IX	X	XI	XII	XIII	XIV	XV	Total	Expansion intensity (*EI*)
1992	Number	5	4	12	7	4	6	5	1	6	4	12	1	4	0	1	72	1% (1992–1997)
	Area	53	46	28	29	34	25	27	18	31	48	57	16	34	0	31	477	
1997	Number	5	6	12	10	2	8	8	3	8	13	12	2	8	1	1	99	14% (1997–2002)
	Area	86	30	33	18	38	19	25	24	37	42	61	31	28	4	29	505	
2002	Number	6	4	15	10	3	10	12	2	12	21	21	5	10	3	1	135	5.7% (2002–2007)
	Area	173	52	87	54	66	43	44	39	44	57	79	60	72	21	39	930	
2007	Number	4	5	20	12	7	11	16	5	15	33	25	8	13	4	1	179	6.1% (2007–2012)
	Area	261	58	104	72	78	55	67	51	82	77	113	79	84	24	41	1246	
2012	Number	7	10	41	17	15	23	29	14	33	53	37	12	18	5	1	315	9.7% (2012–2018)
	Area	349	64	146	98	60	67	97	68	108	132	188	108	127	45	43	1700	
2018	Number	14	12	47	21	20	27	46	16	43	58	37	19	26	10	1	597	
	Area	434	82	343	171	124	98	147	199	167	157	266	164	184	69	81	2686	

The number and area of urban built-up areas has gradually increased in northern region over the past 27 years, while they only started accelerating after 2007 in southern region (Fig 4), suggesting a different temporal pattern of urbanization between these two regions. This might have been caused by the construction of the railroads and expressway. The expressway and railway started operating about from 2005 to 2010 in southern region, respectively. After that, improvement of transportation system promoted connectivity and population mobility between South Xinjiang and other China’s places, hence accelerating economic development and urbanization process [43,44]. On the contrary, the modern transportation system, especially in expressway and railway, in northern were built earlier than those in southern regions, resulting in that northern has been urbanizing continuously over the past 27 years [44].

Expansion intensity (*EI*) represents the relative change of urban built-up area over a given period of time [25]. In this study, our results showed that *EI* values were smallest from 1992–1997 (1%), whereas were moderate in 2002–2007 (5.7%), 2007–2012 (6.1%) and 2012–2018 (9.7%), and was the highest in 1997–2002 (14%) (Table 2). This indicated that the expansion of urban built-up area own a significant feature of periodicity. Similar with other cities in northwest China, the expansion of urban built-up area maintained at relatively slow speed due to a undeveloped economic base from 1992 and 1997 [45]. After that, the stimulation of social and economic recovery of China and the implementation of “Great Western Development Strategy” caused a rapid expansion of urban built-up area from 1997 to 2002 [46]. Between 2003 and 2018, the trade-offs among population growth, the infrastructure investment, the increases in resource consumption, and the implementation of “Supportive Policy of Xinjiang's Development”, promoted a medium expansion of urban built-up area in Xinjiang [47,48].

**Fig 5:**
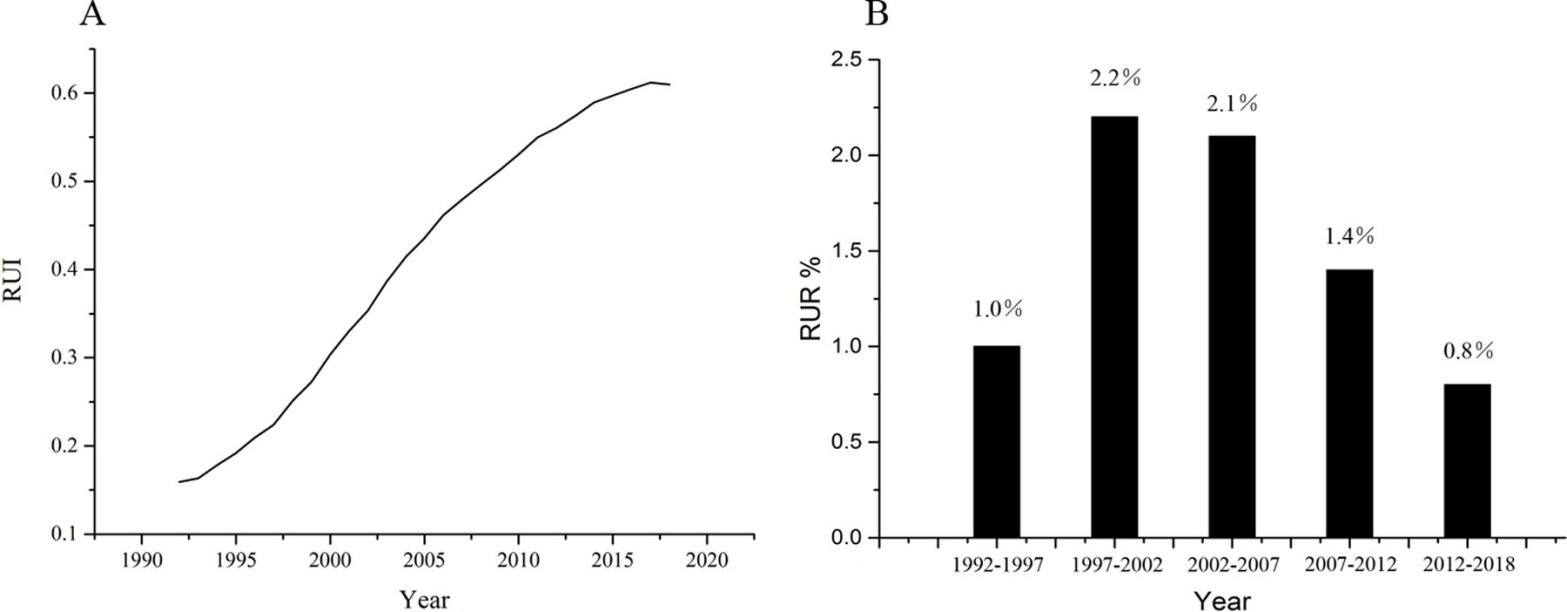
Changes in urbanization index (*RUI*) and urbanization rate (*RUR*) in the Xinjiang Uygur Autonomous Region from 1992 to 2018.

Northam et al., considered that urbanization can be divided into three stages [49], where *RUI* shows the “*S*” shaped curve over time: (1) the initial stage has a low urbanization level and slow urban development; (2) the accelerated stage has an increased urbanization level and fast urban development; and (3) the decelerated stage has a high urbanization level and slow urban development. In this study, *RUI* followed an “*S*” shaped curve from 1992 to 2018 (Fig 5). Based on previous studies regarding urbanization level classification, *RUI*<20%, 20% <*RUI*< 60% and *RUI* >60% were taken as the initial, the accelerated and the decelerated stages, respectively [39,50]. Our results showed that 1992–1997, 1997–2012 and 2012–2018 could be classified as the initial, the accelerated, and the decelerated stages of urbanization, respectively (Fig 5). Interestingly, same results also appeared in changes of *RUR*. Our results showed that *RUR* was 1.1%, 2.2%, 2.1%, 1.4% and 0.8% in 1992–1997, 1997–2002, 2002–2007, 2007–2012 and 2012–2018, respectively (Fig 5). *RUR* was higher in 1997–2012 than in 1992–1997 and 2012–2018 (Fig 5), suggested that urbanization followed the “*S*” shaped pattern. Urbanization rate increased from 1992–2012, while decreased from 2012–2018 in the Xinjiang Uygur Autonomous Region.

**Fig 6:**
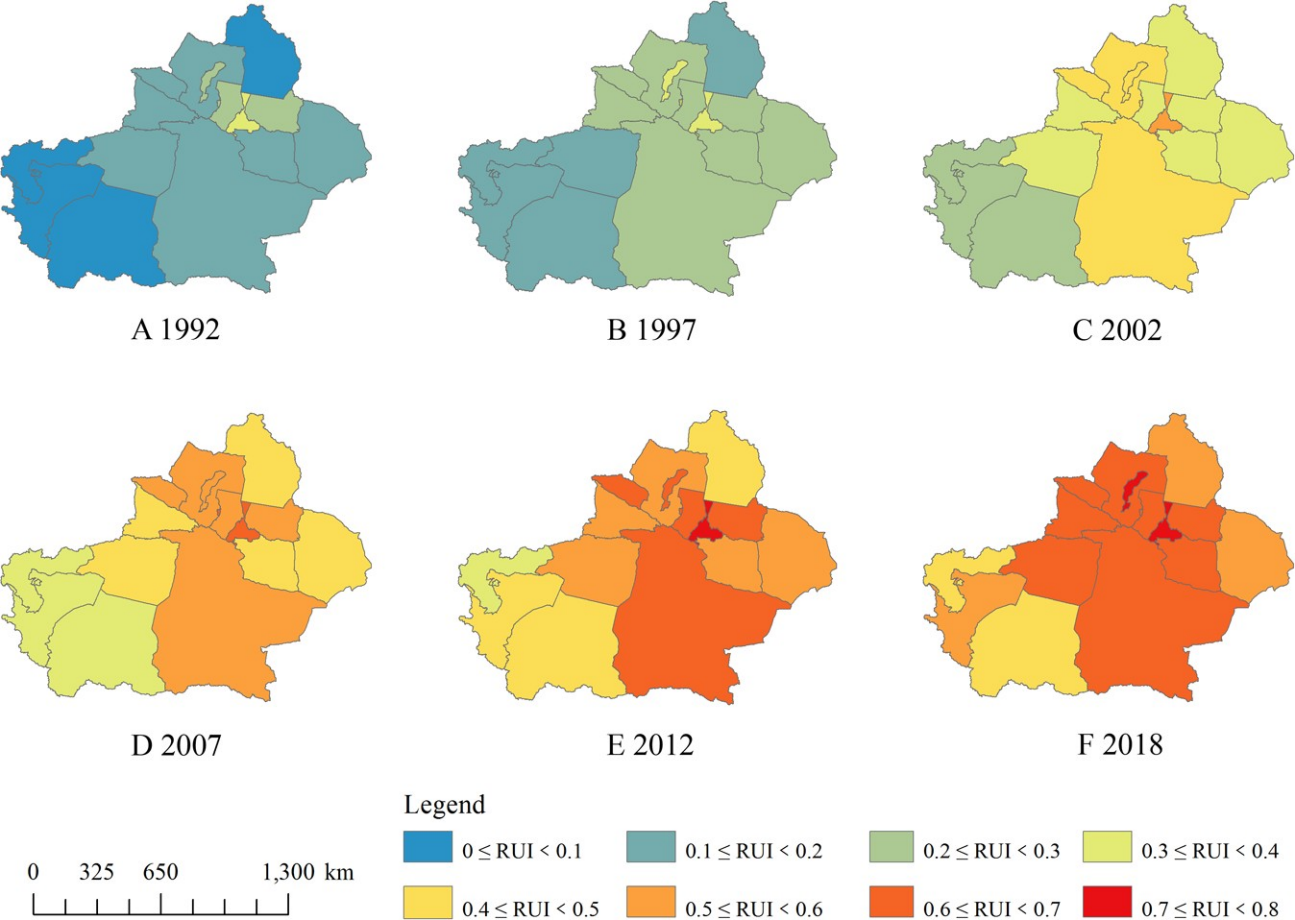
Changes in urbanization index (*RUI*) among different regions in the Xinjiang Uygur Autonomous Region over the past 27 years. Copyright statement as suggested in Fig 1.

The expansion of urban built-up area and *RUI* in large cities were higher than those in small and medium-sized cities (Table 2 and Fig 6). For example, Urumqi (I) had the largest *RUI* among all cites (Fig 6). The number of small cities (with an area of more than 20 km^2^) only increased about 2.31 times from 1992 to 2018, while large cities expanded over 4 times (Fig 6). This suggested that the expansion of urban built-up area of large cities played a decisive role in urbanization of the Xinjiang Uygur Autonomous Region. This is probably because big cities have owned a complete infrastructure, a convenient transportation and the vast majority of employment opportunities [44,48]. The advantages of these factors promoted a continuous expansion of urban built-up areas through large foreign investment, population migration, and high-end technology import [45]. Among all small and medium-sized cities, the Altay region (VI) and the Kizilsu Kirghiz autonomous prefecture (XIV) owned the smallest *RUI* over the past 27 years (Fig 6), probably may be determined by geographical limitation [25,51]. High elevation and the jagged terrain were the most prominent features of these two places. An unfriendly geographical environment are not conducive to urban expansion and industrial and agricultural production, thus maintaining the low urbanization level [25].

### The influences of anthropogenic and geographic environmental factors on urbanization

**Fig 7:**
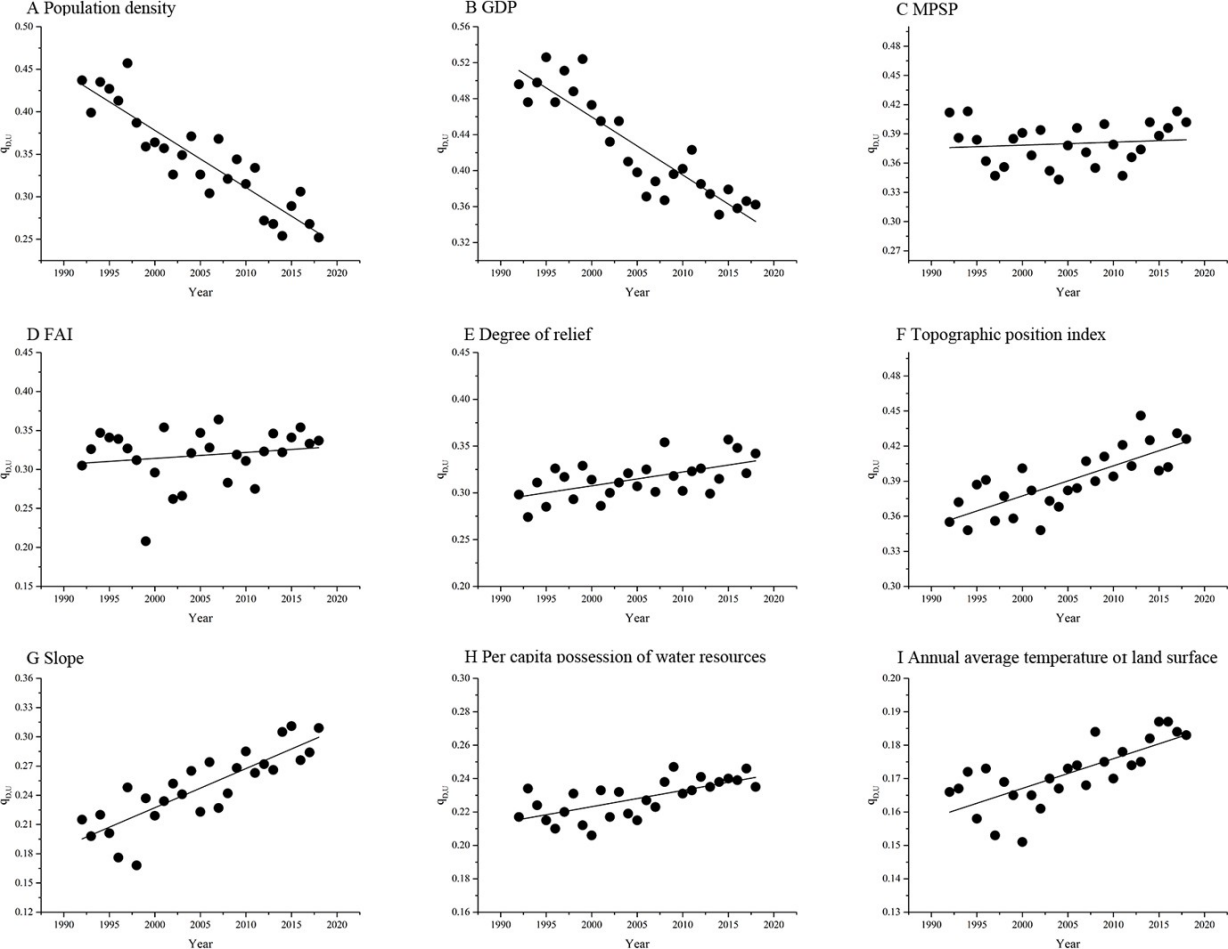
The influences of anthropogenic and geographical environmental factors on urbanization index (*RUI*) in the Xinjiang Uygur Autonomous Region over the past 27 years.

Our results showed that *q*_*D*,*U*_ was greater than 0 for all influencing factors (Fig 7), suggesting that all factors affected urbanization in the Xinjiang Uygur Autonomous Region. Among these factors, GDP and MPSP were considered the two most important factors affecting urbanization [52,53]. This makes sense because the promotions of GDP and MPSP required a continuous increase in urban built-up areas and high urbanization level [52,53]. For example, local governments concentrated formerly scattered industries, manufacturing and producer services into larger industrial parks to improve GDP and MPSP [54]. This form of agglomeration economy promoted urbanization [52–54]. Population density also had a significant influence on urbanization [55]. This was because population density positively correlated with the quantity and area of residential, commercial, industrial and production lands [40,55]. The increase in population density enlarged urban built-up areas, thus promoting urbanization. FAI also had a positive impact on urbanization [56]. In order to continuously develop economy and to upgrade industrial infrastructure, Chinese government introduced many policies, such as the "Great Western Development Strategy", the "Pairing-assistance Strategy" and the "Silk-Road strategy", to promote the development of the Xinjiang Uygur Autonomous Region [57–59]. These policies attracted a large number of funds from society to build industrial parks, commercial strip and infrastructure projects, which thus promoted urbanization in the Xinjiang Uygur Autonomous Region [57–59]. Topographic environmental factors also played an important role in urban expansion and urbanization because they limited the availability of suitable sites for urban constructions [13]. For example, urban buildings were always constructed in places with a superior geographical environment, such as the places with low topographic position index, low degree of relief and slope, high per capita possession of water resources and mild annual average surface temperature [24,25].

**Fig 8:**
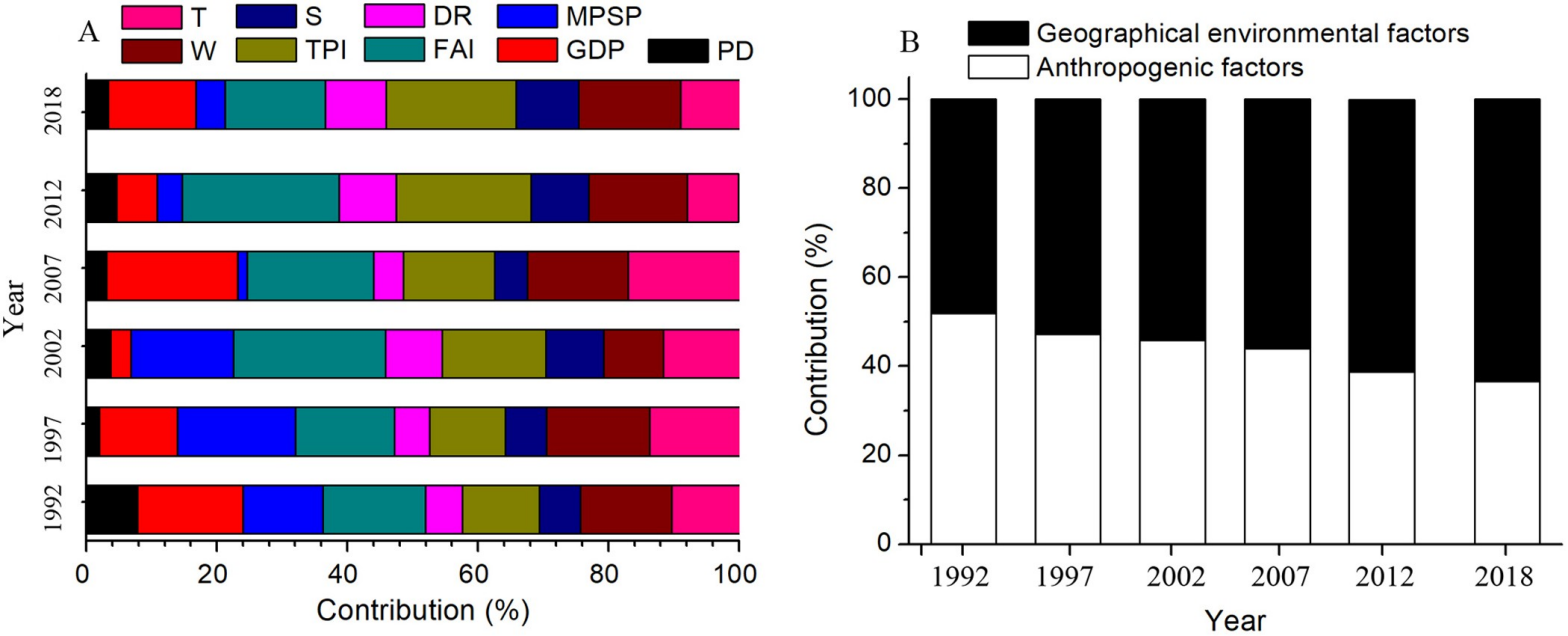
The contributions of anthropogenic and geographical environmental factors to urbanization index (*RUI*) in the Xinjiang Uygur Autonomous Region over the past 27 years.

The influences of population density and GDP on urbanization index (*RUI*) decreased gradually over the past 27 years, whereas that of MPSP and FAI remained stable, but that of degree of relief, topographic position index, slope, per capita possession of water resources and annual average surface temperature continually increased (Fig 7). After classifying these factors into two types: anthropogenic and geographic environmental factors, our results suggested that the influence of anthropogenic factors on urbanization decreased over the past 27 years, while the impact of geographic environmental factors increased (Figs 7 and 8). This indicated that there has been a shift in the influencing factors of urbanization, gradually changing from anthropogenic to geographic environmental factors. This may be related to the geographic location and harsh environmental conditions. Xinjiang Uygur Autonomous Region is the furthest inland region from ocean worldwide. Low rainfall and high evaporation made this region extremely dry. In addition, the Kunlun Mountains, Tianshan Mountains and several deserts (i.e. Taklimakan Desert and Gurbantunggut desert) caused Xinjiang’s terrain more complex. These unfriendly geographical conditions resulted in lower population density and weaker public transportation systems [55]. Compared with other regions of China, Xinjiang’s geographic environment has a lower carrying capacity for economic development [42]. Since the implementation of China’s reform and opening policy after 1980s, the continuous economic development has led to rapid urbanization, however, this extensive development also created a series of problems, especially in mineral resource exploitation [40]. As time has passed, the accumulation of these problems continuously reduced the carrying capacity of geographic environment, which has gradually become the main factor in restricting economic development [24]. As suggested by Northam et al., the “*S*” shaped development model of urbanization indicated that the rapid promotion of urbanization by anthropogenic factors was followed by a decelerated stage where the impact of anthropogenic factors declined [49]. This conclusion also can be demonstrated by the results of the hierarchical partitioning analysis. Our results found that the contribution of anthropogenic factors to urbanization gradually decreased over the past 27 years, while the contribution of geographic environment factors increased (Fig 8). In 1992, anthropogenic and geographic environmental factors contributed 51.9% and 48.1% to urbanization, while in 2018 they contributed 36.6% and 63.4% to urbanization, respectively (Fig 8).

Over the past 27 years, the average contributions of FAI and TPI to urbanization were 18.75% and 15.62%, respectively. Their contributions were obviously higher than other influencing factors (Fig 8), indicating that FAI and TPI were the most important contributors to urbanization. This may be because FAI was directly related to the industrial layout of local governments [60]. Due to the abundant mineral and tourism resources, Xinjiang government built several industrial and tourist cities to improve economy, which resulted in a significant promotion of urbanization [61]. This result differed from many studies that found that GDP and MPSP were the most important factors affecting urbanization in other mainland cities [52]. This difference may be attributed to varying industry types and economic development models [53]. Xinjiang's economy was dominated by state-led mining and tourism, while other mainland cities were dictated by the market [61]. TPI is the average of the elevation difference between the center point and its surrounding area [25]. Compared with other geographic environmental factors, TPI had the highest correlation with convenient transportation and construction cost, hence, was the most important determinant of site selection in urban buildings [25]. Therefore, it has been the largest contributor to urbanization among geographic environmental factors (Fig 8). Interestingly, per capita possession of water resources also obviously affected urbanization. Our result showed that the contribution of per capita possession of water resources to urbanization was 14.18%, and it has the second highest profit among geographic environmental factors (Fig 8). This was probably because Xinjiang is located in an inland arid region, where water is one of the most important limiting factors for population migration, industrial and agricultural production and urbanization [42,62,63].

## Conclusions

In this study, we proposed a new modified thresholding method that combined advantages of both the traditional empirical thresholding and the statistical comparative methods to extract built-up areas from nightlight imageries. Our method has a higher accuracy in the extraction of urban built-up areas than the traditional methods, but the improvement had an obvious difference between DMSP/OLS and VIIRS/DNB nightlight imageries. Over the past 27 years, the expansion pattern of urban built-up areas has an obvious difference between northern and southern regions, as well as between small and large cities in the Xinjiang Uygur Autonomous region. All anthropogenic and geographic environmental factors affected urbanization. There has been a shift in the influencing factors of urbanization, gradually changing from anthropogenic to geographic environmental factors over the past 27 years.

## Supporting information

S1 Data (DOCX)
