# Supplementary material for: Evaluating the influencing factors of urbanization in the Xinjiang Uygur Autonomous Region over the past 27 years based on VIIRS-DNB and DMSP/OLS nightlight imageries
Source: PLoS One. 2020 Jul 22;15(7):e0235903. doi: 10.1371/journal.pone.0235903 (PMC7375535; doi:10.1371/journal.pone.0235903)
Supplement: S1 Data — (DOCX) [file pone.0235903.s001.docx]

**Supplementary Material**

**Method statement**

In the study named “Evaluating the influencing factors of urbanization in Xinjiang Uygur Autonomous Region over the past 27 years based on VIIRS/DNB and DMSP/OLS nightlight imageries”, the modified thresholding method was used to extract all nightlight imageries from 1992 to 2018. But, due to the different sensor, the extraction of built-up urban area might not be consistent between DMSP/OLS and VIIRS/DNB data sources. Thus, the 2012 and 2013 nightlight imageries were used to find some revised method to increase the consistency and validity of the extracted built-up urban areas results from the two different data sources. Here we selected 2012 and 2013 as the references because their nightlight imageries were repeatedly collected in both DMSP/OLS and VIIRS/DNB data sources. The details are listed as following:

1. Based on the constructive process of our modified thresholding method, the thresholds of DMSP/OLS images in 2012 and 2013 were calculated as 60 and 58, while thresholds of VIIRS/DNB images were 22.67 and 21.89, respectively. The coincidence rate between two data sources were 31.2% and 29.4%, respectively (Fig. 1a and Fig. 2a), indicating that the extraction of built-up urban area was lower consistency between DMSP/OLS and VIIRS/DNB images. Here coincidence rate is an assessment indicator to describe the consistency of the extraction of urban built-up areas between two different data sources. The calculation is as follows. The urban built-up areas in 2012 and 2013 were extracted from two data sources by using our modified thresholding method, respectively. Then, the number of same pixels in two extracted urban built-up areas was obtained by comparative method. The ratio of the number of same pixels to the total pixel number in the extracted urban built-up areas from DMSP/OLS images is defined as coincidence rate. Higher coincidence rate indicated larger consistency of the extracted built-up urban area between DMSP/OLS and VIIRS/DNB images.
2. It is generally known that deserts, lakes and high attitude area (>3000 m above sea level, we counted the known elevations of the coordinates of 74 town points, all of which are less than 3000m, so we assume that areas with an altitude of more than 3000m are non-urban areas.) are non-urban areas in the Xinjiang Uygur Autonomous Region. Thus, we removed those non-urban areas from the VIIRS/DNB images, and calculated coincidence rate. The results showed that the thresholds of VIIRS/DNB images changed from 22.67 and 21.89 into 22.39 and 21.53 for 2012 and 2013, respectively. The coincidence rate were raised to 45.2% and 41.3% for 2012 and 2013, respectively (Fig. 1b and Fig. 2b), indicating that the remove of non-urban areas increased the consistency of extraction of built-up urban area between two data resources.
3. Additionally, it has been reported that normalized vegetation index (NDVI) can be used to estimate the ranges of urban built-up areas due to the negative correlation between them (Knight, and Voth 2011; Salmon et al., 2011; Yang et al., 2012; Bo et al., 2015). The area with DNVI greater than or equal to 0.45 was considered as the non-urban area (Wan et al., 2015). In this study, based on the treatments of deserts, lakes and high attitude area, we also removed the non-urban areas where NDVI >0.45 in order to improve the consistency of extraction of built-up urban area between two data resources. The NDVI data uses NDVI product data obtained by MODIS satellites with a resolution of 1 km^2^. Our results showed coincidence rates achieved 74.6% and 70.3% for 2012 and 2013, respectively (Fig. 1c and Fig. 2c), which was higher than that of previous treatments. Therefore, the remove of deserts, lakes, high attitude areas and NDVI >0.45 areas can use to be a revised method in reducing the errors in the extraction of built-up urban area between DMSP/OLS and VIIRS/DNB images. In this study, we used this revised method to treat all VIIRS/DNB images from 2013-2018.

**
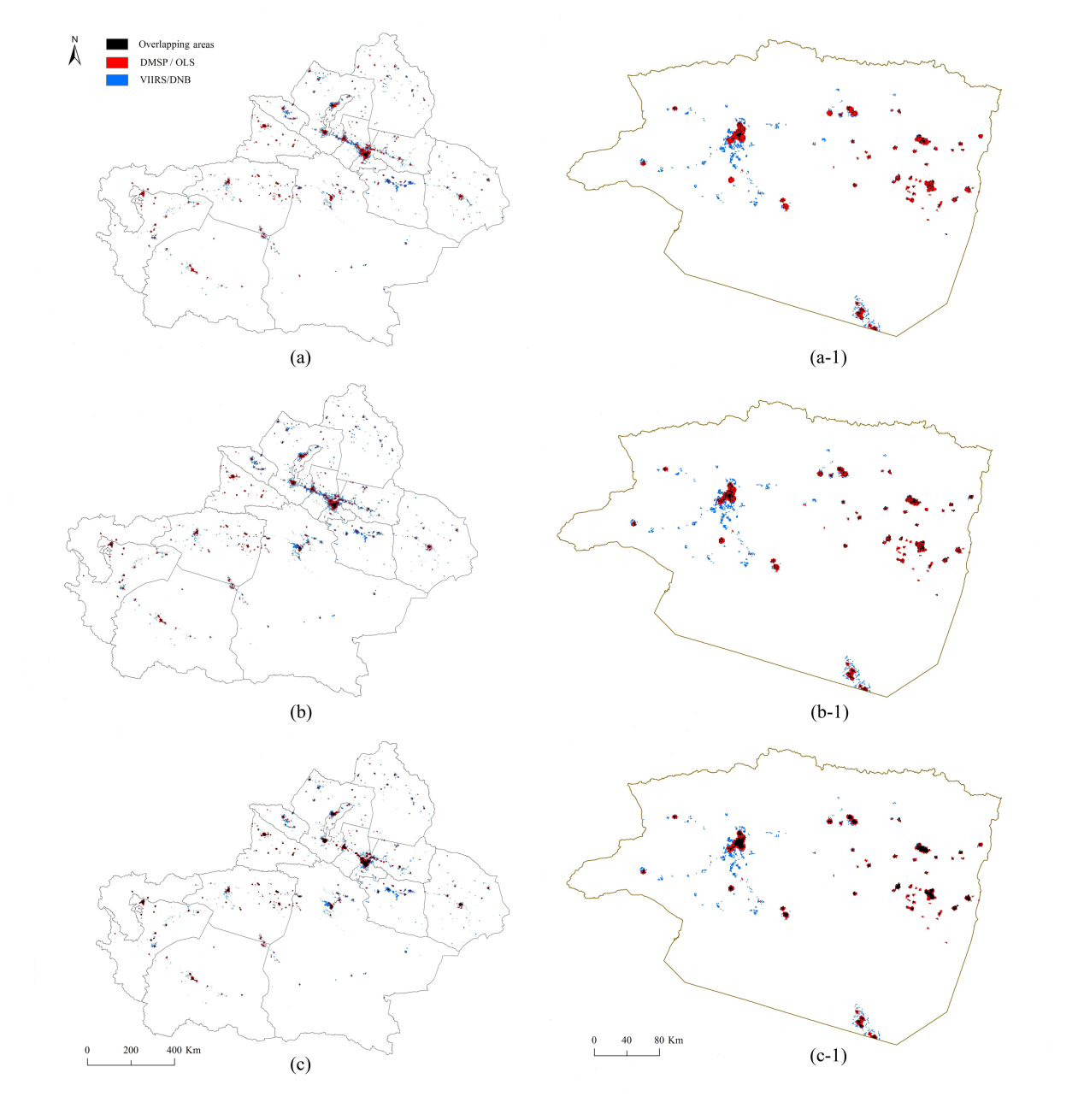
**

**Fig. S1** The changes in coincidence rate of the extracted urban built-up areas between three treatments in 2012. (a): original; (b) the remove of deserts, lakes and high attitude area (>3000 m above sea level); (c) the remove of the remove of deserts, lakes, high attitude areas and NDVI >0.45 areas. At the whole boundary scale of the Xinjiang Uygur Autonomous Region, it is difficult to find the changes in coincidence rate between different treatments due to the small area of spot. Thus, a prefecture-levels city (Aksu) was specially extracted from the diagram of whole Xinjiang to show the differences in coincidence rate among three treatments.


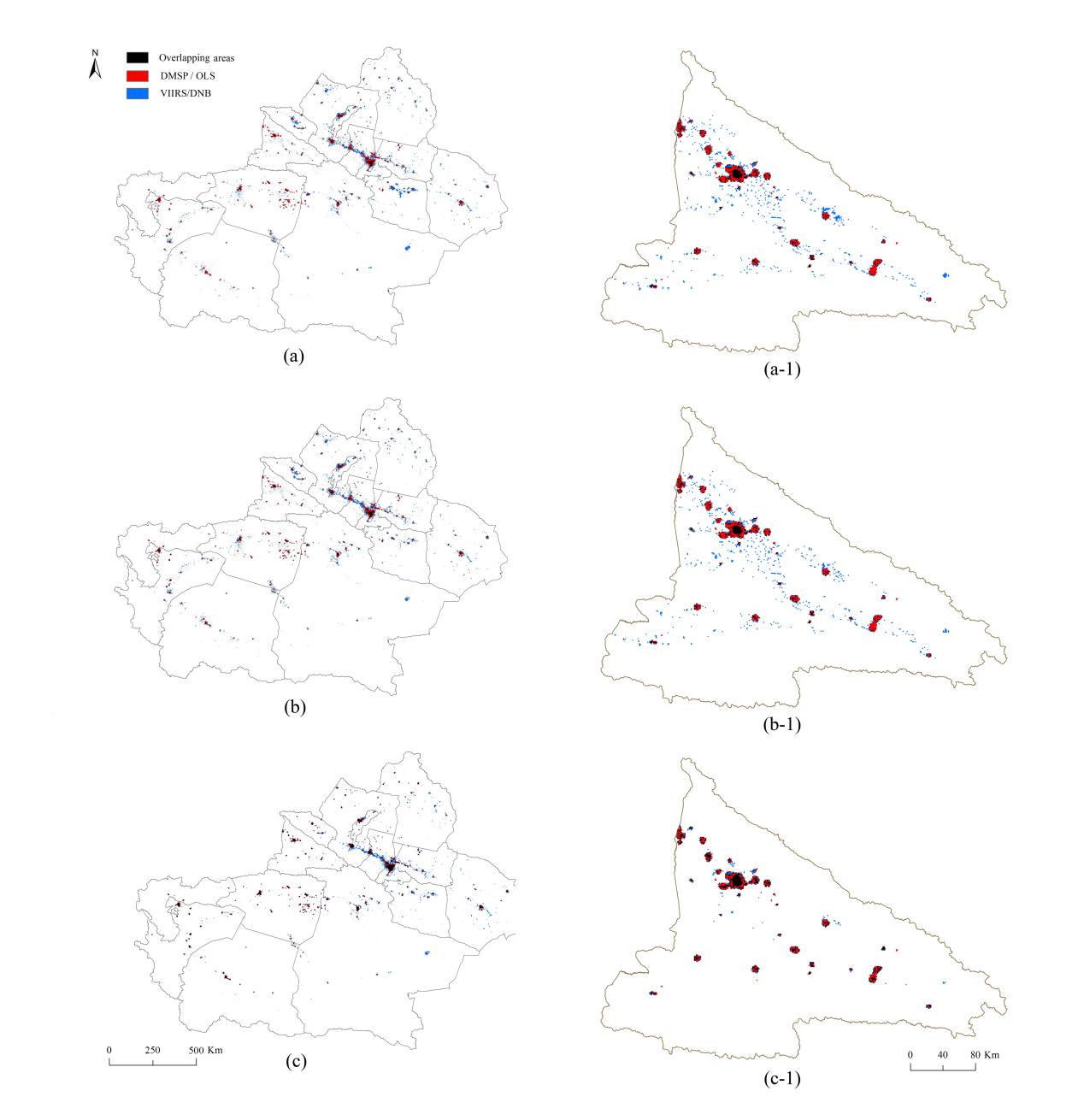


**Fig. S2** The changes in coincidence rate of the extracted urban built-up areas between three treatments in 2013. (a): original; (b) the remove of deserts, lakes and high attitude area (>3000 m above sea level); (c) the remove of the remove of deserts, lakes, high attitude areas and NDVI >0.45 areas. At the whole boundary scale of the Xinjiang Uygur Autonomous Region, it is difficult to find the changes in coincidence rate between different treatments due to the small area of spot. Thus, a prefecture-levels city (Ili) was specially extracted from the diagram of whole Xinjiang to show the differences in coincidence rate among three treatments.

**References**

1. Knight J , Voth M . Mapping Impervious Cover Using Multi-Temporal MODIS NDVI Data. IEEE Journal of Selected Topics in Applied Earth Observations and Remote Sensing. 2011, 4(2):303-309. doi:10.1109/JSTARS.2010.2051535.
2. Salmon B P , Olivier J C , Kleynhans W , Wessels K J , van den Bergh F , Steenkamp K C . The use of a Multilayer Perceptron for detecting new human settlements from a time series of MODIS images. International Journal of Applied Earth Observations & Geoinformation, 2011, 13(6):0-883. doi:10.1016/j.jag.2011.06.007.
3. Yang F , Matsushita B , Fukushima T , Yang W . Temporal mixture analysis for estimating impervious surface area from multi-temporal MODIS NDVI data in Japan. ISPRS Journal of Photogrammetry and Remote Sensing. 2012, 72(none):90-98. doi:10.1016/j.isprsjprs.2012.05.016.
4. Bo W , Qinghua G , Fang F . Mapping US Urban Extents from MODIS Data Using One-Class Classification Method. Remote Sensing. 2015, 7(8):10143-10163. doi:10.3390/rs70810143.
5. Wan B , Guo Q , Fang F , Su Y , Wang R . Mapping Impervious Surface Distribution with Integration of SNNP VIIRS-DNB and MODIS NDVI Data[J]. Remote Sensing, 2015, 7(9):12459-12477. doi:10.3390/rs70912459.
